# Correction: Medaka as a model for human nonalcoholic steatohepatitis

**DOI:** 10.1242/dmm.050367

**Published:** 2023-07-05

**Authors:** Toshihiko Matsumoto, Shuji Terai, Toshiyuki Oishi, Shinya Kuwashiro, Koichi Fujisawa, Naoki Yamamoto, Yusuke Fujita, Yoshihiko Hamamoto, Makoto Furutani-Seiki, Hiroshi Nishina, Isao Sakaida

There were errors in *Dis. Model. Mech.* (2010) **3**, dmm002311 (doi:1242/dmm.002311).

All semi-quantitative RT-PCR data for *SREBP-1c*, *ACC1*, *FAS*, *PPARA*, *CPT1*, *ACO1* and *β-actin* shown in the original Fig. 3E and Fig. 4E represent cropped images of a single set of gels. However, during figure assembly, some images were cropped incorrectly, leading to discrepancies among the images shown.

Specifically, there were errors in assembly of the *SREBP-1c* (Fig. 4E, top), *FAS* (Fig. 3E, left), *CPT1* (Fig. 4E, bottom) and *β-actin* (Fig. 3E, left; Fig. 4E, top and bottom) gels. The authors have provided the corrected versions of these panels as a single figure. All citations to Fig. 3E and Fig. 4E in the main text should now refer to this new panel.

**Fig. 3E/4E (corrected panel).**
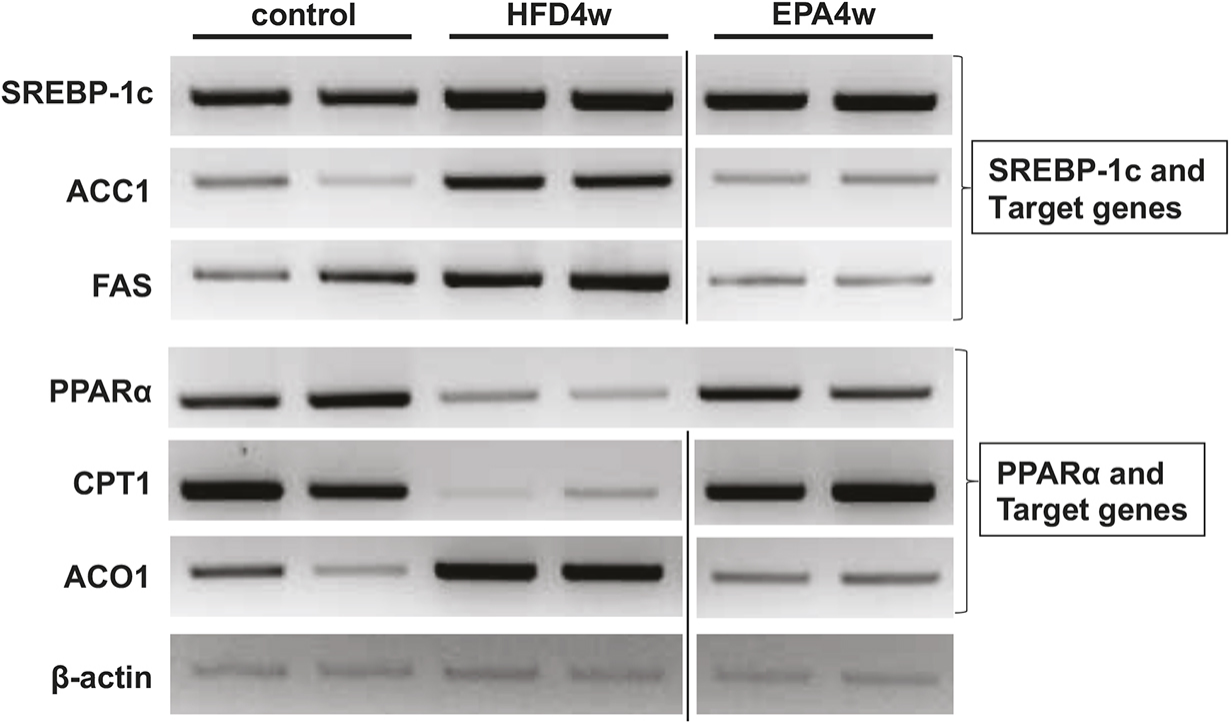
(E) Altered expression of lipogenic and lipolytic genes. mRNA levels of the indicated genes in livers from control (lanes 1 and 2), HFD-medaka (lanes 3 and 4), and HFD+EPA medaka (lanes 5 and 6) at 4 weeks were determined by semi-quantitative RT-PCR. *SREBP-1c*, *ACC1* and *FAS* were all elevated in HFD-medaka compared with their levels in control medaka. Conversely, the mRNA levels of *PPARA* and *CPT1* were reduced and those of *ACO1* were increased. Compared with HFD-medaka, the mRNA levels of *SREBP-1c*, *ACC1* and *FAS* were reduced in HFD+EPA medaka. Conversely, the expression levels of *PPARA* and *CPT1* were increased in HFD+EPA medaka, whereas the level of *ACO1* was decreased. This pattern also resembled that observed in medaka that were fed the control diet (*n*=6/group).

This correction does not affect the results in the article or the conclusions of this study. The authors apologise for these errors and any inconvenience they may have caused.

